# Genes within the serotonergic system are differentially expressed in human brain

**DOI:** 10.1186/1471-2202-10-50

**Published:** 2009-05-15

**Authors:** Karen Sugden, Ales Tichopad, Nadeem Khan, Ian W Craig, Ursula M D'Souza

**Affiliations:** 1 MRC Social, Genetic and Developmental Psychiatry (SGDP) Centre, Institute of Psychiatry, King's College London, UK; 2 Physiology Weihenstephan, Center of Life and Food Sciences (ZIEL), Technical University of Munich, 85350 Freising, Germany; 3 Department of Neuropathology, Brain Bank, Institute of Psychiatry, King's College London, UK

## Abstract

**Background:**

Serotonin is an important neurotransmitter with wide-ranging functions throughout the central nervous system. There is strong evidence to suggest that regulation of serotonergic gene expression might be related to genetic variability, and several studies have focused on understanding the functional effects of specific polymorphisms within these genes on expression levels. However, the combination of genotype together with gender and brain region could have an overall effect on gene expression. In this study, we report expression patterns of five serotonergic genes (*TPH1*, *TPH2, 5-HT2A*, *5-HT2C*, *5-HTT*) in seven different human post-mortem brain regions (superior frontal gyrus, superior temporal gyrus, striatum, cerebellum, hippocampus, midbrain and thalamus) using TaqMan™ real-time quantitative PCR. In addition, the effect of genotype and gender on their expression levels was determined.

**Results:**

The data revealed that mRNA from the five genes investigated was detected in all brain regions and showed an overall significant difference in expression levels. Furthermore, the expression of *5-HT2C*, *5-HT2A *and *TPH2 *was found to be significantly different between the various brain regions. However, neither gender nor genotype showed significant effects on the expression levels of any of the genes assayed. Interestingly, *TPH1 *and *TPH2 *were expressed in all brain regions similarly except for within the striatum and cerebellum, where *TPH1 *was expressed at a significantly higher level than *TPH2*.

**Conclusion:**

The effect of brain region has a greater influence on serotonergic gene expression than either genotype or gender. These data add to the growing body of evidence that effects of functional polymorphisms on gene expression *in vitro *are not observed *ex vivo*, and provide information that will aid in the design of expression studies of the serotonergic gene system within human post-mortem brain.

## Background

Serotonin (5-HT) is an important neurotransmitter found in neurons which project to various areas in the central nervous system. Consequently it is associated with several physiological mechanisms [1]. It mediates cellular effects through several proteins that are involved with its neurotransmission, synthesis, metabolism and membrane re-uptake [2,3]. Disturbances in brain serotonergic systems result in a range of phenotypes such as depression, suicide and anxiety disorders [3-5].

Many functional imaging and binding studies have demonstrated that these disorders can, in part, be explained by differential binding and/or function of components of the serotonergic system such as receptors and enzymes. However, whether these observed differences correspond to differences in gene expression is largely unknown. Regulation of expression occurs via several different processes, but most studies have focused on genetic variation as a potential factor [6]. However, although many polymorphic variations have been demonstrated to elicit changes in expression *in vitro *(studies in cell cultures), little information on their role in *ex vivo *investigations using human post-mortem brain tissue exists. Furthermore, despite much attempt to determine a link between 5-HT pathway gene polymorphisms and disease phenotype, few consistent findings have been made including the promoter polymorphism in the *5-HTT *gene and anxiety-related traits [7,8]. Although there are many methodological and biological reasons for this, one explanation might be that differential regulation could be elicited via additional factors such as tissue type and gender as well as background genetic variation.

Some of the most widely studied serotonergic genes include tryptophan hydroxylase 1 and 2 (*TPH1 *and *TPH2; *serotonin synthesis), serotonin receptors 2A and 2C (*5-HT2A *and 5-*HT2C*; post-synaptic receptors) and serotonin transporter (*5-HTT*, serotonin re-uptake). TPH is the rate-limiting enzyme in the biosynthetic pathway of serotonin and thus has a major function in regulating the serotonergic system. Studies in mice have revealed the existence of two isoforms of the gene termed as *TPH1 *and *TPH2*, with rat and human homologues of the second isoform also cloned [9]. Although the discovery of *TPH2 *is relatively recent, evidence has emerged in support of its expression in human post-mortem brain. Firstly, *TPH2 *expression was observed in specific post-mortem brain regions including cortex, thalamus, hippocampus, amygdala and hypothalamus [10]. Secondly, differential expression of *TPH1 *and *TPH2 *genes in various post-mortem brain regions has been demonstrated, with *TPH2 *expression being significantly higher in raphe nuclei [10]. However, *TPH1 *expression was found to be significantly higher than that of *TPH2 *in hypothalamus and amygdala. The functional effects of genetic variation in *TPH1 *and *TPH2 *genes have had little investigation. However, there is evidence that in the *TPH1 *gene the TGT haplotype of 3 SNPs (T-1607C, G-1066A, T-346G) within the 5'regulatory region repressed transcriptional activity in human cell lines [11]. Similarly, [12] have demonstrated that a haplotype of 3 SNPs within the *TPH2 *promoter (G-703T, T-473A, and A90G) also influenced transcription in rat and human cell lines, the TTA haplotype resulting in reduced gene expression.

5-HT2A and 5-HT2C receptors are both expressed post-synaptically on serotonergic neurons. 5-HT2A receptors are found mainly in the cortex and hippocampus [13,14] whilst 5-HT2C receptors are found in greatest density in the choroid plexus, although they are also located in other brain regions such as the prefrontal cortex [15,16]. It has been suggested that there is some similarity between the neuronal distribution of 5-HT2A binding sites and *5-HT2A *mRNA [14]. More recently, quantitative RT-PCR has revealed more abundant 5-*HT2A *mRNA in the cortex compared with the hippocampus in rat brain [17]. Interestingly, 5-HT2A receptor density has been shown to be under gender-specific influence, where men demonstrated significantly higher binding capacity of receptors than women [18].

There are a number of polymorphisms in the 5-*HT2A *gene, some of which have been investigated in terms of their effect on transcription, particularly the two highly-linked SNPs T102C and A-1436G. Both the A and G alleles of the latter have significant basal promoter activity in HeLa cells [19]. More recently, the transcriptional activity of the G allele was found to be significantly lower than the A allele when the more downstream of the two promoters was included in the constructs and in the presence of an enhancer region [20]. This effect was only observed in endogenous 5-*HT2A *expressing cell-lines, suggesting the presence of cell specific transcription factors is necessary to elicit this functional effect. However, no differences were found in allele-specific expression of the A-1436G SNP in human post-mortem brain tissue [21].

Four variants within the promoter of the *5-HT2C *gene have been identified and their influence on gene expression investigated [22]. Two haplotypes each consisting of a particular allele of an upstream GTn microsatellite (Z-6) were found to increase expression of the gene relative to the wild-type. Furthermore, this effect was not cell-specific, suggesting that it would be present in any tissue expressing *5-HT2C*.

5-HTT is responsible for re-uptake of serotonin at the pre-synaptic neuron, and is the site of action of several selective serotonin reuptake inhibitors and tricyclic antidepressants. The gene is expressed widely in the brain with highest expression detected in the dorsal raphe nuclei [23]. One of the most documented variants within *5-HTT *is the serotonin transporter-linked polymorphic region (5-HTTLPR), a repeat region which exists as two common alleles, the short allele ('S') and long ('L'). There is evidence that this polymorphic region has functional significance, where in human carcinoma cells and lymphoblastoid cells, the L allele confers higher activity than the S allele [24]. More recently, it has been shown that the S and L alleles are themselves variable in that SNPs occur within their repeating units [25]. Another functional VNTR (variable number of tandem repeat) within intron 2 of the *5-HTT *gene has revealed differential enhancer activities in the mouse embryo [26] and embryonic stem cells [27].

Here, we focused on first determining whether the mRNA expression levels of *TPH1, TPH2, 5-HT2A, 5-HT2C *and *5-HTT *genes are affected by brain region, genotype or gender. Real-time quantitative PCR (qPCR) was utilised to determine the relative expression levels for these genes in seven different human post-mortem brain regions. The investigated polymorphisms are described above and are located either within, or close to, the postulated promoter region of the genes of interest suggesting a plausible functional effect. Given the extensive literature concerning the significance of *TPH1 *in disease aetiology, and the potential complication of the existence of its paralogue *TPH2*, the second aim was to compare the levels of expression of the two loci in the various brain regions.

## Results

**Figure 1:**
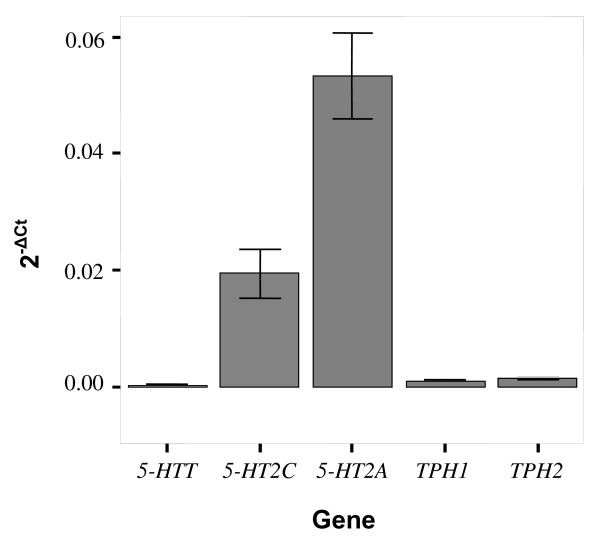
**Bar graph representing the expression (2^-ΔCt^) of five serotonergic genes before controlling for brain region, genotype or gender**. All genes are expressed in the brain, *5-HTT *showing the lowest expression and *5-HT2A *the highest. Bars represent mean and error bars represent standard error of the mean (s.e.m).

All genes assayed were expressed in all brain regions tested, and these data are represented in Figure 1. Preliminary analyses of the relative expression across all brain regions (grouping individual 2^-ΔCt ^values) showed an overall significant difference in the mRNA levels of the five genes investigated (p < 0.001 F = 108.782). *5-HT2A *has an overall highest expression level, whereas *5-HTT *had the lowest expression level.

### Effect of gender on gene expression

**Figure 2:**
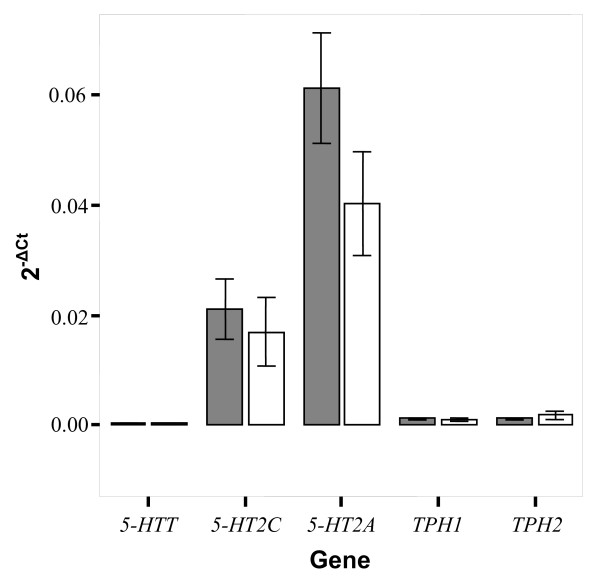
**Bar graph representing the effect of gender on expression (2^-ΔCt^) of the five genes in all brain regions**. Males (grey bars) and females (white bars) represent the mean and error bars representing s.e.m. No significant differences in expression are observed.

Gender did not have any significant effect on the overall brain expression levels of the genes we assayed in this sample (data combined for all seven brain regions). However, expression of most genes was generally higher in males than in females (see Figure 2). Furthermore, there were no significant gender differences on gene expression levels within any of the brain regions we assayed (data not shown).

### Effect of brain region on gene expression

**Figure 3:**
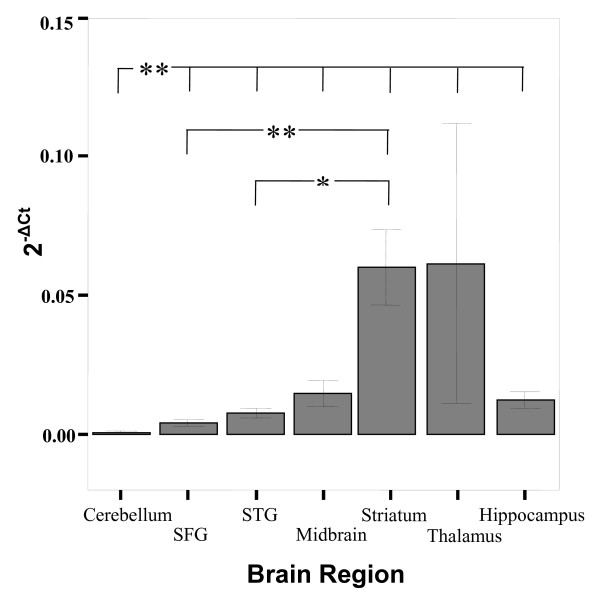
**Bar graph describing the expression (2^-ΔCt^) of *5-HT2C *in various brain regions**. There are significant differences in expression (denoted by ** [p < 0.001]) across the brain regions, mainly due to lower expression of genes in the cerebellum. The symbol * represents a significant difference P = 0.003 between *5-HT2C *expression in striatum and STG brain regions (see Table 1). Bars represent the mean and error bars represent s.e.m. The groups representing expression in the thalamus have N = 2.

**Figure 4:**
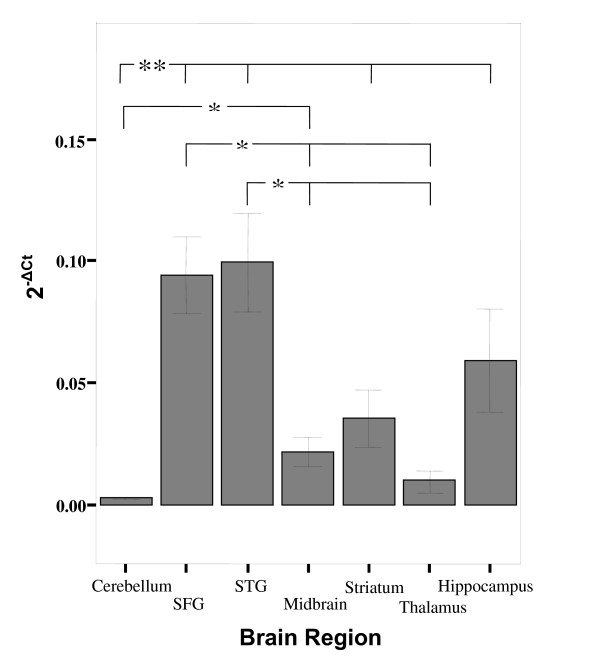
**Bar graph describing the expression (2^-ΔCt^) of *5-HT2A *in various brain regions**. There are significant differences in expression (denoted by ** [p < 0.001] and * [p ≤ 0.04]) across the brain regions, mainly due to lower expression of genes in the cerebellum (see Table 1). Bars represent the mean and error bars represent s.e.m.

**Figure 5:**
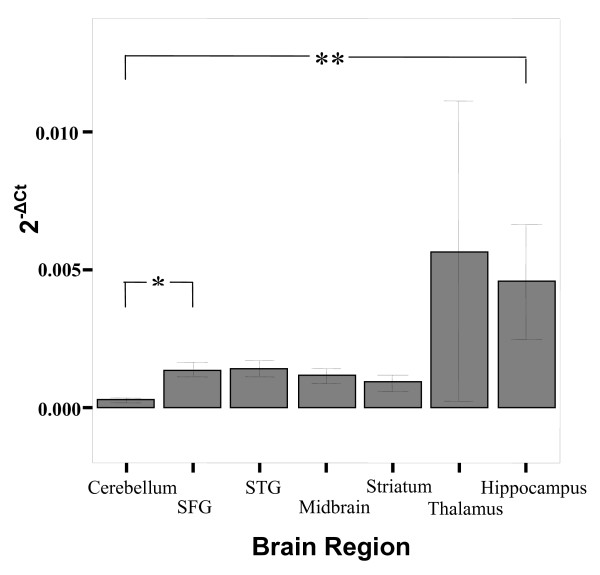
**Bar graph describing the expression (2^-ΔCt^) of *TPH2 *in various brain regions**. There are significant differences in expression (denoted by ** [p = 0.001] and * [p = 0.011]) across the brain regions, mainly due to lower expression of genes in the cerebellum (see Table 1). Bars represent the mean and error bars represent s.e.m. The groups representing expression in the thalamus have N = 2.

There were significant differences in expression levels of three genes within different brain regions. These genes included *5-HT2C *(F = 19.297, p < 0.001), *5-HT2A *(F = 17.952, p < 0.001) and *TPH2 *(F = 4.294, p = 0.001), and are represented in the bar charts of Figure 3, 4 and 5, respectively. The significant differences were consistently described by lower expression in the cerebellum compared to other brain regions as shown in Table 1.

**Table 1:** Results of comparisons of gene expression levels for those genes showing differences between brain regions

Gene	Brain region 1	Brain region 2	Mean Difference (1–2)	Std. Error	Bonferroni-corrected *p *value	95% Confidence Intervals	
*5-HT2C*	CEREBELLUM	SFG	-0.779	0.176	0.001	-1.334	-0.223
		STG	-1.041	0.179	<0.001	-1.606	-0.476
		STRIATUM	-1.748	0.173	<0.001	-2.295	-1.201
		MIDBRAIN	-1.180	0.210	<0.001	-1.843	-0.518
		THALAMUS	-1.835	0.358	<0.001	-2.965	-0.705
		HIPPOCAMPUS	-1.338	0.247	<0.001	-2.117	-0.559
	STRIATUM	SFG	0.969	0.170	<0.001	0.432	1.506
		STG	0.706	0.173	0.003	0.159	1.253
*5-HT2A*	CEREBELLUM	SFG	-1.430	0.164	<0.001	-1.948	-0.911
		STG	-1.427	0.164	<0.001	-1.946	-0.909
		MIDBRAIN	-0.706	0.196	0.012	-1.324	-0.088
		STRIATUM	-0.934	0.170	<0.001	-1.471	-0.397
		HIPPOCAMPUS	-1.267	0.230	<0.001	-1.994	-0.541
	SFG	MIDBRAIN	0.724	0.193	0.008	0.113	1.335
		THALAMUS	0.905	0.279	0.039	0.023	1.788
	STG	MIDBRAIN	0.721	0.193	0.008	0.111	1.332
		THALAMUS	0.903	0.279	0.040	0.021	1.785
*TPH2*	CEREBELLUM	SFG	-0.746	0.203	0.011	-1.392	-0.101
		HIPPOCAMPUS	-1.205	0.277	0.001	-2.087	-0.323

Mean differences were calculated where relative expression values of brain region 2 were subtracted from the value of brain region 1. The significance levels and 95% confidence levels are listed for comparisons that show significant differences only.

### Effect of genotype on gene expression

Genotype frequencies and results of ANOVA of the effects of genotype on gene expression are shown in Table 2. As 5-*HT2C *is localised to the X-chromosome, we split the sample by gender when assessing genotype effects. *TPH1 *did not show any variation in genotype (all samples were "GG" after successful digestion, N = 11), and so no further analyses could be performed for this gene. In general, there were no significant effects of genotype on overall brain expression of any of the genes that were analysed. There appears to be a dose-dependant effect on expression of the *5-HT2A *gene by A-1438G genotype (mean 2^-ΔCt ^± S.D.; AA genotype = 0.041 ± 0.048, AG genotype = 0.048 ± 0.052, GG genotype = 0.054 ± 0.065; ANOVA F = 0.02, p = 0.983). However, this failed to reach significance. Due to the small numbers in the groups stratified by genotype and allele frequency, further analysis of the effects of polymorphisms on within-brain region expression could not be reliably performed.

**Table 2:** Results of expression analyses by genotype

						**H-W test **		**ANOVA**	
**Gene/Marker**		**Genotype**	**Genotype** ** Frequency** **(*N*)**	**Allele**	**Allele** **Frequency** **(*N)***	** χ^2 ^value**	***P***	***F***	***P***
***5-HT2A***		AA	0.29 (4)	A	0.46 (13)	0.57	0.75	0.02	0.983
A-1438G		AG	0.36 (5)	G	0.54 (15)				
		GG	0.36 (5)						
		N = 14							
***5-HT2C***									
promoter microsatellite	M	Z	0.67 (4)					0.02	0.98
		Z-6	0.33 (2)						
	F	Z-6/Z-6	0.14 (1)	Z-6	0.36 (5)	0.84	0.89	0.14	0.933
		Z-6/Z	0.29 (2)	Z	0.57 (8)				
		Z-6/Z+2	0.14 (1)	Z+2	0.07 (1)				
		Z/Z	0.43 (3)						
		N = 13							
***TPH2***		GG	0.69 (9)	G	0.85 (22)	0.94	0.33	1.47	0.23
G-703T		GT	0.31 (4)	T	0.15 (4)				
		N = 13							
***5-HTT***		SS	0.07 (1)	S	0.39 (11)	0.43	0.81	0.10	0.91
5-HTTLPR		SL	0.64 (9)	L	0.61 (17)				
		LL	0.29 (4)						
		N = 14							
***5-HTT***		10/10	0.09 (1)	10	0.23 (5)	0.72	0.70	0.02	0.98
Intron 2 VNTR		10/12	0.27 (3)	12	0.77 (17)				
		12/12	0.64 (7)						
		N = 11							

Genotype frequencies, allele frequencies, results of H-W (Hardy-Weinberg) tests and ANOVA comparisons of gene expression levels (log_(10) _2^-ΔCt^) values for the polymorphic variants are shown. Genotypes of the *5-HT2C *marker are split by gender (Males = M; Females = F) since the males are hemizygotic.

### *TPH1 *vs. *TPH2 *expression

**Figure 6:**
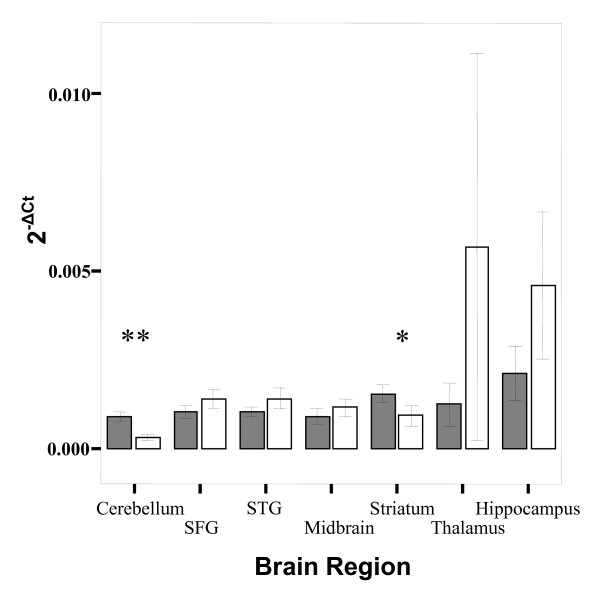
**Bar graph of mean expression (2^-ΔCt^) of *TPH1 *(grey bars) versus *TPH2 *(white bars) and error bars representing s.e.m**. Both genes are expressed in all brain regions, and *TPH1 *expression is significantly higher than *TPH2 *in both the striatum (t = 2.487, p = 0.022, denoted by *) and cerebellum (t = 4.297, p < 0.001, denoted by **). The groups representing *TPH2 *expression in the hippocampus and thalamus have a much larger variance than that of *TPH1*, probably due to the smaller number of individuals in these groups (hippocampus N = 5, thalamus N = 2).

Overall expression of *TPH1 *is not significantly different to that of *TPH2 *in this sample (t = 1.613, p = 0.101, mean 2^-ΔCt ^± S.D.; *TPH1 *= 0.001 ± 0.0008, N = 68; *TPH2 *= 0.001 ± 0.002, N = 63). However, when the data were further analysed for each brain region the expression of *TPH1 *was significantly higher than *TPH2 *in the striatum (t = 2.487, p = 0.022) and cerebellum (t = 4.297, p < 0.001) (See Figure 6). There appear to be differences in the expression of *TPH1 *and *TPH2 *in the hippocampus and thalamus but as only a small number of data points were obtained for these brain regions, no significant differences could be determined. In addition, no significant differences exist between the relative expression of *TPH1 *and *TPH2 *when stratified by gender (data not shown).

As the possible presence of Alzheimer's disease at death was recorded for 8 individuals, we tested for its possible impact on transcription. However, there was no significant influence of this phenotype on the difference in gene expression levels in all brain regions tested (*TPH1*, t = 1.33, d.f. = 13, p = 0.21, *TPH2*, t = 0.62, d.f. = 13, p = 0.55; *5-HT2A*, t = 2.17, d.f. = 8, p = 0.06; *5-HT2C*, t = 0.85, d.f. = 13, p = 0.41; *5-HTT*, t = 2.04, d.f. = 13, p = 0.06).

## Discussion

This is the first investigation, to our knowledge, that compares the expression patterns of different serotonergic genes in various human post-mortem brain regions. The results suggest that all five serotonergic genes are expressed in human post-mortem brain tissue and significant overall differences in expression of *5-HT2A*, *5-HT2C *and *TPH2 *were observed between the various brain regions analysed. This is in agreement with previous findings that show variation in receptor densities between brain regions [28-30], and suggests that these differences in protein levels might be determined by transcriptional regulation to some degree. Differences in abundance of these various serotonergic transcripts throughout the brain suggest differential gene regulation between specific brain regions. Therefore, it is important to take into consideration the brain region from which the mRNA is derived when performing comparative post-mortem brain expression studies.

Perhaps most surprisingly, there appeared to be little effect of genetic variation on the overall brain expression of the genes we analysed. Although it has been suggested that expression of all five genes is regulated by promoter polymorphisms *in vitro *[11,12,20,22,31], we did not observe the same effect *ex vivo*. Similar results have been observed in previous expression studies in human post-mortem brain. For example, differences in *5-HT2A *expression were not observed between the 'A' and 'G' allele of the 5-*HT2A *A-1438G SNP [21], and allelic ratios of *5-HTT *mRNA are not correlated to *5-HTTLPR *genotype in human pons [32] or brainstem [33]. Whilst our data are in agreement with these findings, interpretation should be with some caution due the small sample size for each genotype group. This limits our ability to determine any brain region-specific effects of genetic variation on gene expression. Therefore these findings will need to be confirmed by using a larger sample-size in future studies. In addition, other potential confounders that were not measured could have an effect. For example, there has been a report of *5-HT2A *imprinting in human fibroblasts and brain [34], although other independent studies found no further evidence for this phenomenon [21,35]. Overall, the data described here suggest that the postulated functional polymorphisms do not affect gene expression and this may reflect the complexity of gene regulation *ex vivo*.

No effects of gender on expression levels were observed for any of the genes within this sample. Interestingly, little data exist to date regarding the effect of gender on the expression of human serotonergic genes. However, there is evidence to suggest that gender-specific hormones might exert some regulatory effect on expression in rodents and primates. In Rhesus macaques, administration of oestrogen and progesterone resulted in a decrease in expression of *5-HT2C *in the hypothalamus [36], whilst treatment of rats with oestradiol lead to a decrease in *5-HT2C *expression in the hippocampus [37]. Subsequently, the expression of both *TPH1 *and *TPH2 *was shown to increase in the dorsal raphe nuclei of both mice [38] and macaques [39] following oestradiol administration. In addition, both testosterone and oestrogen were capable of increasing the number of 5-HTT cells in castrated male rats [40]. A limitation of the observations of our studies regarding the situation in humans is that the tissue donors were elderly, and reports of the normal levels of sex hormones within this age group are conflicting [41,42].

Finally, the results observed here reveal that *TPH1 *expression is detected in all investigated brain regions at a comparable or higher level than that of *TPH2*. These data confirm recent findings [10], where *TPH1 *expression was observed in human brain, and that expression of *TPH1 *was significantly higher than that of *TPH2 *in some of the brain regions we also examined (frontal cortex, thalamus, hippocampus, hypothalamus and amygdala). Our data show that *TPH1 *expression is higher than that of *TPH2 *in the striatum and cerebellum, but not the hippocampus. This inconsistency could be related to variables we could not measure, or factors such as the differing mean ages of samples analysed in this study and that by Zill and colleagues [10], since there is evidence that increasing age enlarges the heterogeneity of gene expression contributing to greater inter-individual variability in expression [43]. Additionally, the discrepancy in our results with that of Zill and colleagues could be due to the existence of transcriptional variants for TPH2 mRNA in humans. Recent evidence in rats has revealed that TPH2 has two transcriptional variants one of which is expressed at a very low level [44]. The potential of TPH2 mRNA splice variants has not been demonstrated in humans to date and warrants future investigation.

The original discovery of the novel *TPH2 *isoform led to the hypothesis that it was *TPH2 *not *TPH1 *that was the relevant 5-HT catabolic gene in brain tissue based on its much higher expression level (150 times more) in the brain stem of mice [9]. This region contains raphe nuclei which are rich in serotonergic neurons and our results differ from these previous studies as we focused on several other brain areas which did not include the brain stem. Prior to discovery of *TPH2*, brain regional differences in TPH densities were traditionally explained by differences in translational efficiencies of splice variants [45]. However, this might now be explained by region specific expression of the two *TPH *isoforms. Our data support this hypothesis as *TPH1 *is expressed in all brain regions, and that there are brain region specific differences in expression of *TPH1 *and *TPH2*. Although emerging data suggest that TPH1 is not sufficient to drive serotonergic metabolism in the brain, it is expressed, albeit at differing levels, and as such does not preclude its role in the regulation of serotonergic functioning. Furthermore, TPH1 in the pineal gland is essential in the synthesis of melatonin, a mechanism by which it can, in turn, influence serotonergic functioning and behaviour [46].

Although we attempted to control for factors that could have an effect on results described here, there are other confounding issues to consider. Firstly, the sample size is small, which might lead to bias in group comparisons. Furthermore, specific conditions at the time of death and post-mortem (e.g. Post mortem interval, PMI) significantly affect RNA integrity and abundance [47-50]. However, these studies show that post-mortem intervals of 48 hours do not affect expression levels. The PMI of the post-mortem samples utilised in this study were 38.67 ± 16.62 hours (mean ± S.D.) and within the limit so that gene expression levels would not be affected.

Real-time qPCR ('TaqMan') was utilised to quantify gene expression since it is a highly reproducible and sensitive technique [51,52]. The relative quantification method of analysis was employed which compares the expression level of a target gene to the expression of an endogenous control gene. This high-throughput method has been successfully used in previous gene expression studies with post-mortem human brain and biopsies [53,54]. However, there are some caveats to be made regarding choice of suitable endogenous control gene, especially in regards to expression stability. In this study, *SDHA, UBC *and *GAPDH *housekeeping genes were used as endogenous controls based on previous data which showed these genes to be most stably expressed in human neuroblastoma cells [55]. The results did not reveal any significant differences between relative expression values when normalised to each of the endogenous control genes separately (data not shown), thus differential expression of control gene is unlikely to be a confounding factor.

## Conclusion

This investigation has demonstrated the differential expression of 5-HT genes in several human post-mortem brain tissue regions using real-time qPCR. These data provide additional information that will be relevant for appropriate sample selection when performing gene expression studies involving human brain. Both gender and transcriptionally-relevant polymorphisms appear to have little influence on *ex vivo *expression within this sample suggesting that complex interactions present in human brain tissue might eclipse the genetic effects that are consistently observed *in vitro*. These results, taken together with those from alternative approaches such as animal models, *in vitro *and neuroimaging studies, will contribute to the understanding of these interactions and inform researchers of the mechanisms that regulate serotonergic gene expression in human brain.

## Methods

The sample consisted of brain tissue collected from 15 individuals post-mortem (9 Male, 6 female) provided by the Maudsley Brain Bank (Department of Neuropathology, Institute of Psychiatry, London, UK) and stored at -70°C prior to use. These individuals were normal (N = 7) adults and Alzheimer's patients (N = 8) with agonal states of either cardiac failure, bronchopneumonia, myocardial infarction, hypertension, coronary occlusion, carcinoma of left kidney, ischaemic heart disease, pulmonary embolus. Mean age at death for the sample was 78.0 ± 7.4 years (mean ± s.d.), and mean post-mortem delay was 38.7 ± 16.6 (mean ± s.d.) hours. No significant differences were observed between males and females with regard to either age at death (males: 77.6 ± 7.0 years (mean ± s.d.); females: 79.2 ± 8.6 years (mean ± s.d.) t = -0.381, p = 0.712) or post-mortem delay (males 38.2 ± 14.1 hrs (mean ± s.d.); females 39.3 ± 21.3 hrs (mean ± s.d.); t = -0.112, p = 0.913). From each individual, samples from up to 7 different brain regions were collected and included the following: superior frontal gyrus (SFG; N = 15), superior temporal gyrus (STG; N = 15), hippocampus (N = 7), cerebellum (N = 15), midbrain (N = 9), thalamus (N = 3) and striatum (N = 14). The Ethical Committee (Research) at the Institute of Psychiatry considered and approved this experimental project and the reference study number is 167/00.

### Nucleic acid preparation

Brain tissue was stored at -80°C prior to total RNA and DNA extraction using TRI reagent (Sigma, UK) following the manufacturer's instructions. Total RNA was DNase I treated using Qiagen RNeasy kit (Qiagen, UK) and DNase I (Qiagen, UK) following manufacturer's recommendations. cDNA was generated from total RNA using the TaqMan RT-PCR kit (Applied Biosystems, UK). Briefly, 1 μg of total RNA was reverse transcribed with 5.5 mM of MgCl, 500 μM of each dNTP, 2.5 μM random hexamers and 0.4 U/μL RNase inhibitor in a total volume of 20 μl. Samples were incubated at 25°C for 10 minutes, followed by 48°C for 30 minutes and then denatured at 95°C for 5 minutes in a thermocycler.

### Relative quantitative real-time PCR using 'TaqMan'

Primers and probes for each gene were obtained via 'Assays-on-demand' (Applied Biosystems, UK). All probes were tested for equal reaction efficiencies prior to use by making a serial dilution of a single cDNA sample (range 1–10^3 ^dilution). All probes showed an amplification efficiencies in the accepted range (0.98 – 1.02). Assay ID numbers are listed in Table 3. All probes are designed to span adjacent exons to minimise effects of any residual DNA contamination.

**Table 3:** Details of qPCR and genotyping assay primers and conditions

**Gene**	**Assay on demand ID**	**Genotype variant**	**Genotype detection method**	**Genotyping primers (forward and reverse)**	**Size (bp)**	**Temp** **°C**	**MgCl_2_** **(mM)**
***TPH1***	Hs00188220_m1	T-346G	*MslI *enzyme digest	5'CTTCGTTATGTGTACAGTCC-3' 5'TAGGACTGCAGTGCTTCTC-3'	G = 365 T = 305+60	59	2
***TPH2***	Hs00542783_m1	G-703T	Taqman allelic discrimination	5'ACACTCACACATTTGCATGCAC-3' 5'CATTGACCAACTCCATTTTATGTTAATAAGCT-3'			
				*TPH2 *taqman genotyping probe sequences: VIC-CTTGACATATTCTAATTTT FAM-ACTTGACATATTATAATTTT			
***5-HT2A***	Hs00167241_m1	A-1438G	*MspI *enzyme digest	5'AAGCTGCAAGGTAGCAACAGC-3' 5'AACCAACTTATTTCCTACCAC-3'	A = 468 G = 244+224	56	1.5
***5-HT2C***	Hs00168365_m1	GTn promoter microsatellite -1027 bp	capillary electrophoresis	FAM5'GGGAGTTTCAAAGCTTGATGA3' 5'GTTTCTTAGACCCATGGTGGAGATGG-3'	~259; Z-6 allele	59.5	2.5
***5-HTT***	Hs00169010_m1	*5-HTTLPR*	agarose gel electrophoresis	5'ATGCCAGCACCTAACCCCTAATGT-3' 5'GGACCGCAAGGTGGGCGGGA-3'	419; 16 rpt 'L'	66	1.5
		intron2 VNTR	agarose gel electrophoresis	5'GTCAGTATCACAGGCTGCGAG-3' 5'TGTTCCTAGTCTTACGCCAGT-3'	STin12 ~299	54	1.5
***GAPDH***	Hs00266705_g1	n/a					
***UBC***	Hs00824723_m1	n/a					
***SDHA***	Hs00188166_m1	n/a					

Assay-on-demand' IDs correspond to product order numbers (Applied Biosystems).

TaqMan reactions were performed using 0.5 μl Assays-on-demand primer/probe, 5 μl 2× TaqMan mastermix (Applied Biosystems, UK), 0.5 μl cDNA and appropriate volume of primer and probe in a total volume of 10 μl. Concurrently, three housekeeping endogenous controls were assayed for each individual sample for normalisation purposes (Succinate Deydrogenase (*SDHA)*, Ubiquitin C (*UBC*) and Glyceraldehyde-3-phosphate dehydrogenase (*GAPDH) *[55]). Each experiment was performed in triplicate. The TaqMan reaction was carried out using an ABI7900HT sequence detection system set up in absolute quantification mode. Cycling conditions were one cycle of 95°C at 10 minutes, followed by 40 cycles of 95°C at 15 sec and 60°C for 1 min. On completion of PCR, Ct values were generated using SDS 2.1. software.

### Genotyping

The following polymorphisms were genotyped: 5-HTTLPR and intron 2 VNTR (*5-HTT*), T-346G (*TPH1*), G-703T (*TPH2*), A-1438G (*5-HT2A*), GTn microsatellite (*5-HT2C*). Details of these markers, PCR reaction conditions and detection methods are given in Table 3. PCR reactions consisted of 2.5 μl × 10 PCR reaction buffer IV (Abgene, UK), 200 mM dNTPS (Abgene, UK), 1 unit Taq polymerase (Promega, UK), 10 pmol each primer, appropriate concentration of MgCl_2 _and 25 ng template DNA up to 25 μl. Cycling conditions consisted of initial denaturation at 94°C for 4 minutes followed by 30 cycles of 95°C for 1 minute, appropriate annealing temperature for 1 minute and 72°C for 1 minute, followed by a final elongation step at 72°C for 4 minutes. PCR reactions were carried out using an MJ research tetrad thermal cycler (MJ research, UK).

Enzyme digests were carried out using *Msp*I (Promega, UK) or *Msl*I (NEB, USA) following manufacturer's instructions. Digested samples were subjected to agarose gel electrophoresis using a 2% agarose gel (Abgene, UK) and visualised using UV transillumination (UVP, UK). Those assays requiring capillary gel electrophoresis were analysed using an ABI3100 (Applied Biosystems) set up in genotyping mode. 1 μl PCR product was prepared in 12 μl HI-DI formamide and 0.4 μl GSRox size standard (Applied Biosystems, UK), denatured at 95°C for 10 minutes and snap cooled on ice. Data were analysed using Genotyper V3.6 software (Applied Biosystems, UK).

### Statistical analyses

Mean triplicate Ct values for each sample were calculated, and outliers within the triplicates removed using Grubb's method. Briefly, if the obtained Z value (the difference between the investigated Ct and the mean Ct divided by the SD) exceeded the critical value 1.15, the sample was considered an outlier and removed from further analysis. The mean Ct values were used to calculate relative expression using the ΔCt method [56] where normalisation using multiple reference genes was employed. The normalisation index was calculated as the arithmetic mean Ct of the three housekeeping genes (*GAPDH, UBC, SDHA*). The ΔCt was then calculated by subtracting the normalisation index from the mean target Ct, and then the relative expression is calculated using the formula 2^-ΔCt^. Relative expression values (2^-ΔCt^) were log-transformed prior to analysis since these values are not normally distributed [57]. Student's *t *test or ANOVA with *post-hoc *Bonferroni correction and Games-Howell test were performed.

The statistical package for social scientists (SPSS, USA) was employed in all analyses, and grouping variables were gender, genotype and brain region.

## Authors' contributions

KS participated in design of the study, carried out sample preparation, qPCR, statistical analyses and drafted the manuscript. AT participated in statistical analyses. NK performed sample collection. IWC participated in design of the study. UMD conceived of the study, participated in its design and coordination, was involved in statistical analyses and helped draft the manuscript. All authors read and approved the final manuscript.
